# Acute murine antigen-induced arthritis is not affected by disruption of osteoblastic glucocorticoid signalling

**DOI:** 10.1186/1471-2474-15-31

**Published:** 2014-02-03

**Authors:** Cornelia M Spies, Edgar Wiebe, Jinwen Tu, Aiqing Li, Timo Gaber, Dörte Huscher, Markus J Seibel, Hong Zhou, Frank Buttgereit

**Affiliations:** 1 Department of Rheumatology and Clinical Immunology, Charité – Universitätsmedizin Berlin, Charitéplatz 1, D-10117 Berlin, Germany; 2 Bone Research Program, ANZAC Research Institute, Concord Repatriation Hospital, The University of Sydney, Hospital Road, Concord Sydney, NSW 2139, Australia; 3 German Rheumatism Research Centre (DRFZ), Charitéplatz 1, D-10117 Berlin, Germany; 4 Berlin-Brandenburg Centre of Regenerative Therapies (BCRT), Charité - Universitätsmedizin Berlin, Augustenburger Platz 1, D-13353 Berlin, Germany; 5 Department of Endocrinology & Metabolism, Concord Repatriation Hospital, Sydney, Australia

**Keywords:** 11-beta-hydroxysteroid dehydrogenase type 2, Disease models, Animal, Arthritis, Glucocorticoids, Osteoblasts

## Abstract

**Background:**

The role of endogenous glucocorticoids (GC) in the initiation and maintenance of rheumatoid arthritis (RA) remains unclear. We demonstrated previously that disruption of GC signalling in osteoblasts results in a profound attenuation of K/BxN serum-induced arthritis, a mouse model of RA. To determine whether or not the modulation of the inflammatory response by osteoblasts involves T cells, we studied the effects of disrupted osteoblastic GC-signalling in the T cell-dependent model of antigen-induced arthritis (AIA).

**Methods:**

Acute arthritis was induced in pre-immunised 11-week-old male 11β-hydroxysteroid dehydrogenase type 2 transgenic (tg) mice and their wild-type (WT) littermates by intra-articular injection of methylated bovine serum albumine (mBSA) into one knee joint. Knee diameter was measured every 1–2 days until euthanasia on day 14 post injection. In a separate experiment, arthritis was maintained for 28 days by weekly reinjections of mBSA. Tissues were analysed by histology, histomorphometry and microfocal-computed tomography. Serum cytokines levels were determined by multiplex suspension array.

**Results:**

In both short and long term experiments, arthritis developed in tg and WT mice with no significant difference between both groups. Histological indices of inflammation, cartilage damage and bone erosion were similar in tg and WT mice. Bone volume and turnover at the contralateral tibia and systemic cytokine levels were not different.

**Conclusions:**

Acute murine AIA is not affected by a disruption in osteoblastic GC signalling. These data indicate that osteoblasts do not modulate the T cell-mediated inflammatory response via a GC-dependent pathway.

## Background

It is unknown whether and how *endogenous* glucocorticoids (GC) contribute to the initiation and maintenance of rheumatoid arthritis (RA) and other inflammatory diseases [1-7]. We previously studied the role of endogenous GC in the K/BxN serum-induced arthritis mouse model of RA [8]. This model is T cell-independent as arthritis is elicited by antibodies even if the recipients are devoid of lymphocytes [9,10]. Immune complexes of arthritogenic anti-glucose-6-phosphate isomerase autoantibodies attract and activate neutrophils and macrophages at the cartilage surface through Fc receptor binding (particularly FcγRIII) and activation of complement factors from the initial part of the alternative complement pathway [11-15]. This induces the release of pro-inflammatory cytokines including interleukin (IL)-1 and tumour necrosis factor-α [16].

We have recently demonstrated that inactivation of endogenous glucocorticoids in osteoblasts by overexpression of the GC-inactivating enzyme, 11β-hydroxysteroid dehydrogenase type 2 (11β-HSD2), results in attenuation of K/BxN serum-induced arthritis [8]. These findings indicated an immunostimulatory role of endogenous glucocorticoids and suggested that osteoblasts modulate the immune-mediated inflammatory response via a GC-dependent pathway. While K/BxN serum-induced arthritis is principally a T cell-independent model of rheumatoid arthritis, osteoblasts and osteoblastic GC-signalling may or may not modulate the T cell-mediated inflammatory response in other models of arthritis. For example, the crosstalk between T cells and osteoblasts is known to be important in intermittent parathyroid hormone induced bone formation, involving Wnt signalling by Wnt10b [17], which we have previously shown to be GC-dependent in osteoblasts during development [18]. Osteoblasts interact with T cells also by the production of cytokines such as IL-6 [19], and IL-6 expression in osteoblasts is likely to be GC-regulated [20].

In order to test whether or not the modulation of the inflammatory response by osteoblasts involves T cells, we studied the effects of disrupted osteoblastic GC-signalling in the T cell-dependent model of antigen-induced arthritis (AIA) [21,22]. In this model, an adaptive immune response is initiated by immunisation against the non-self antigen methylated bovine serum albumine (mBSA). Local re-injection into the knee joint induces a mainly CD4+ T cell-mediated arthritis [21,22].

## Methods

### Transgenic mouse model

Disruption of GC signalling in mature osteoblasts and osteocytes was achieved through transgenic overexpression of the GC-inactivating enzyme, 11β-hydroxysteroid dehydrogenase type 2 (11β-HSD2), under the control of the osteoblast-specific 2.3-kb collagen type Iα1 promotor (Col2.3-11β-HSD2-transgenic mice). Animals were generated [23] and characterised as described previously [18,23-25], and generously provided by Dr Barbara Kream, University of Connecticut, USA). Mice were maintained at the animal facilities of the ANZAC Research Institute, in accordance with Institutional Animal Welfare Guidelines and according to an approved protocol. An ethics approval for the use of animals was obtained from the Sydney Local Health District Animal Welfare Committee.

### Initiation and clinical assessment of antigen-induced arthritis

#### Immunisation

Antigen-induced arthritis (AIA) in mice was induced following established protocols [26,27]. Eight-week-old male Col2.3-11β-HSD2 transgenic (tg) mice and their wild-type (WT) littermates were immunised by subcutaneous injection of 100 μg methylated bovine serum albumin (mBSA) (on day −21) (Sigma, Castle Hill, Australia), dissolved in 50 μl of phosphate-buffered saline (PBS) and emulsified in 50 μl of Freund’s complete adjuvant (CFA) (Sigma), into both flanks (50 μg each). A second injection of the same dose was given 7 days later (on day −14) by subcutaneous injection into the tail base. For control purposes, both groups – arthritic and control animals – were immunised. Mice were randomised to the respective groups matched for body weight and litter.

#### Induction of acute AIA

AIA was induced by intra-articular injection on day 0 in Col2.3-11β-HSD2 tg mice (n = 17) and their WT littermates (n = 17). Mice were anaesthetised with isoflurane (inhalation anaesthetic), and a total of 10 μg mBSA in 5 μl sterile PBS was injected intra-articularly through the patellar ligament into the right knee joint with a Hamilton syringe and a 25^1/2^ gauge needle. Concurrently, tg (n = 14) and WT mice (n = 15) receiving 5 μl of sterile PBS by intra-articular injection, served as controls (CTR). Body weight and knee joint swelling were assessed every 1–2 days from the time of induction (day 0) up to day 14. Knee joint diameter was measured using a Vernier caliper. The maximum medial-to-lateral diameter was defined at the widest point of each knee joint. Knee joint swelling was calculated as the absolute difference to the knee joint diameter measured at baseline before arthritis induction. Mice were euthanised on day 14 for tissue and blood collection. Tissue of n = 5 mice of each group was preserved for future RNA analysis. Histological, microfocal-computed tomography (micro-CT), histomorphometric and serum cytokine analyses were performed in the remaining tg and WT arthritic and control mice, respectively.

#### Induction of prolonged arthritis

Prolonged antigen-induced arthritis was induced in order to study longer-term effects of AIA on arthritis and bone. Since we wished to keep mechanical damage, potentially induced by repeated intra-articular injections, to a minimum, we induced flare-ups by intravenous re-injections of mBSA, as has been described before [26,28]. Following induction of acute AIA in Col2.3-11β-HSD2 tg mice and their WT littermates, arthritic mice received repeated intravenous injections of 300 μg mBSA (in 200 μl PBS) on days 7, 14 and 21 [26,28]. Tg and WT control mice received 200 μl sterile PBS intravenously (n = 10 per arthritic group, n = 9 per control group) at the same time points. Body weight and knee joint swelling were assessed as described above. Mice were sacrificed on day 28. During the flare-up experiment, 8 mice were excluded [5 anaphylactic reactions (all in mBSA-injected mice), 3 mechanical damages (2 in arthritic mice, 1 in control)]. The relatively high frequency of anaphylactic reactions was unexpected [26,28]. The remaining arthritic tg (n = 6) and WT mice (n = 7), and control tg (n = 9) and WT (n = 8) mice were assessed for histological, micro-CT, histomorphometric and serum cytokine analyses.

### Tissue collection and specimen preparation

Blood for serum analyses was collected by cardiac puncture. Right and left knee joints (including tibia and distal femur) of each mouse were dissected and fixed for 48 hours in 4% paraformaldehyde/PBS. After micro-CT analyses, the knees and tibiae were decalcified in 10% ethylenediaminetetraacetic acid (EDTA) and embedded in paraffin. Serial 4-μm sections were stained with haematoxylin and eosin (H&E) for general histological evaluation and with toluidine blue for assessment of cartilage degradation and proteoglycan loss [8]. To identify osteoclasts, sections were stained for tartrate-resistant acid phosphatase (TRAP) using naphthol-AS-MX phosphate (Sigma) as a substrate and fast red violet LB salt (Sigma) as a detection agent for the reaction product [8].

### Histopathological scoring

Sections of knee joints were stained with H&E or toluidine blue or TRAP, and scored by 3 independent and blinded investigators (EW, CS, JT) for synovitis, soft tissue inflammation, joint space exudate, cartilage degradation/proteoglycan loss, and bone erosion, according to an established semi-quantitative scoring system [27].

### Micro-CT

Left tibiae analysis was performed using a SkyScan 1172 scanner (SkyScan, Kontich, Belgium). Scanning was done at 100 kV and 100 μA, using a 1-mm aluminium filter with the exposure set to 590 msec. In total, 1,800 projections were collected at a resolution of 6.93 μm/pixel. Reconstruction of sections was done using a modified Feldkamp cone-beam algorithm, with the beam hardening correction set to 50%. To quantify the trabecular morphometry of the proximal tibia, CTAnalyser software version 1.02 (Sky-Scan) was used. The greyscale index was set from 75 to 255 per cent. 3D-methods were used in the calculation algorithms. The volume of interest was selected within the endosteal borders, 1–2.3 mm below the growth plate [8]. Slide thickness was 7 μm.

### Histomorphometry

Bone histomorphometry of the left proximal tibial metaphysis was conducted in single measurements on 4-μm sections stained with TRAP or H&E, using OsteomeasureXP v.3.2.1.5 (OsteoMetrics, Inc., Decatur, GA, USA). The region of interest was a 1.5 x 1-mm area of cancellous bone located 0.3 mm below the growth plate of the tibia [8]. Osteoclast number and osteoclast surface relative to bone surface were measured (400x magnification). Osteoblasts were identified by morphology (400× magnification).

### Measurement of serum cytokines

Serum levels of tumour necrosis factor α (TNF-α), interferon-γ (IFN-γ), murine interleukin-1α (IL-1α), IL-1β, IL-2, IL-3, IL-4, IL-5, IL-6, IL-9, IL-10, IL-12(p40), IL-12(p70), IL-13, IL-15, IL-17A, IL-18, monocyte chemotactic protein-1 (MCP-1), macrophage inflammatory proteins 1α (MIP-1α), 1β (MIP-1β) and 2 (MIP-2), regulated upon activation normal T cell expressed and secreted (RANTES), keratinocyte-derived cytokine (KC), monokine-induced by interferon-gamma (MIG), eotaxin, leukaemia inhibitory factor (LIF), basic fibroblast growth factor (basic-FGF), platelet-derived growth factor homodimer (PDGF-BB), vascular endothelial growth factor (VEGF), granulocyte colony-stimulating factor (G-CSF), granulocyte macrophage colony-stimulating factor (GM-CSF), and macrophage colony-stimulating factor (M-CSF) were all determined using the cytometric bead array technique [29]. Premixed cytokine assays (Bio-Rad, Munich, Germany) were used according to the manufacturer’s instructions. Data acquisition was conducted using the Bio-Plex suspension system (Bio-Rad). Each sample was read in duplicate and measured against the mean of two dilution rows. For analysis, values below the detection limit were substituted with the value of 50% of the detection limit.

### Statistical analysis

For comparisons of normally distributed data between 2 experimental groups, the *t*-test was used. Unless otherwise stated, values are the mean and standard deviation (SD) or standard error of the mean (SEM). Non-normally distributed data were compared between two groups using the Mann–Whitney test. In the case of planned multiple comparisons for the same parameter multiple two-group comparisons were conducted only if the Kruskal-Wallis test over all 4 groups was significant. Here the Bonferroni correction was used to adjust the significance level to α* = 0.0125 for 4 parallel tests (WT-CTR versus tg-CTR, WT-AIA versus tg-AIA, WT-CTR versus WT-AIA, tg-CTR versus tg-AIA). The clinical results as obtained over the 14-day or 28-day experimental course were modelled by a generalised linear model with repeated-measures analysis. The *P*_AIA_ values given are for the interaction day × group of within-subjects effects from the repeated-measures analysis, indicating differences in the time course of transgenic and WT arthritic mice. IBM SPSS Statistics version 19 (IBM, Armonk, NY, USA) was used for statistical analysis. *P* values of less than 0.05 were considered significant.

## Results

### Acute AIA

#### Body weight

On day 0, prior to the intervention, body weights of WT and tg littermates were similar (mean ± SD 37.6 ± 3.6 g vs. 36.9 ± 3.1 g; *P* = 0.454). From day 0 on, body weight gain over 14 days was similar in arthritic and control mice [*P* = 0.077] as well as in arthritic WT compared with arthritic tg mice [*P*_AIA_ = 0.908].

#### Knee joint swelling

**Figure 1:**
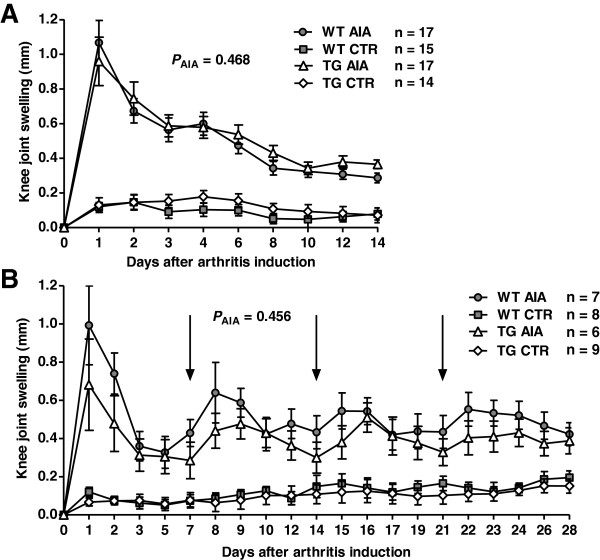
**Clinical assessment of inflammation in arthritic mice injected with mBSA and non-arthritic control mice.** Arthritic mice were injected with mBSA on day 0 (AIA) and control mice were injected with phosphate buffered saline (CTR). **(A)** Means and SEM for knee joint swelling from day 1 to day 14 post intra-articular injection. Knee diameter was measured every 1–2 days and knee joint swelling was calculated as the difference to the knee diameter at day 0 (before arthritis induction) for each day. A repeated-measures analysis was performed (see Materials and Methods); the *P*_AIA_ value indicates the significance of the difference in variation of knee joint swelling over time between wild-type (WT) and transgenic (TG) mice treated with mBSA. **(B)** Means and SEM for knee joint swelling from day 1 to day 28, post intra-articular injection and three flare-up reactions induced by intravenous mBSA injections on days 7, 14, 21 (arrows). Knee diameter was measured every 1–2 days. The *P*_AIA_ value represents the significance derived by repeated-measures analysis between WT AIA mice and transgenic AIA mice.

Knee joint diameter prior to arthritis induction (day 0) did not differ between WT and tg mice (mean ± SD 4.058 ± 0.206 mm and 4.057 ± 0.209 mm, respectively; *P* = 0.979). Both WT and tg mice treated with mBSA developed acute arthritis with significant knee joint swelling in comparison to controls [*P* < 0.001], with maximum values on day 1 post injection (mean increase in joint diameter ± SD: +1.07 ± 0.53 mm and +0.96 ± 0.58 mm, respectively). Following day 1, knee joint swelling resolved over time in both groups. There was no statistically significant difference in knee joint swelling between arthritic WT and tg mice [*P*_AIA_ = 0.468] (Figure 1A).

#### Histopathological assessment of arthritis

**Figure 2:**
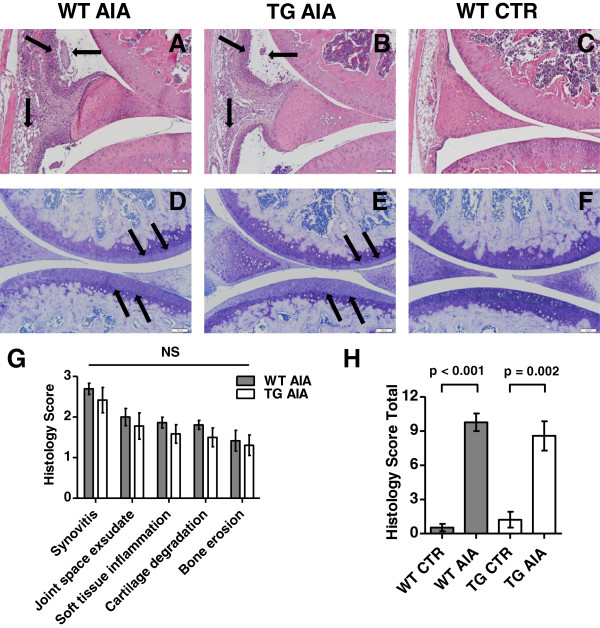
**Arthritis and cartilage damage in the knee joint 14 days post intra-articular injection. (A-F)** Representative histologic sections of knee joints from wild-type (WT) and transgenic mice (TG) treated with mBSA (AIA) and from non-arthritic wild-type control mice (CTR). Both inflammatory activity and cartilage damage were similar in transgenic mice and wild-type mice treated with mBSA. **(A–C)** Hematoxylin and eosin staining. **Arrows** show synovitis, joint space exudate and soft tissue inflammation. Bars = 100 μm. (**D–F)** Toluidine blue staining. **Arrows** show proteoglycan loss of articular cartilage. Bars = 100 μm. (**G-H)** Histopathology scores (**G** single, **H** total) in mBSA-treated mice (AIA) 14 days post injection. The knee joints of all mice were assessed as described in Materials and Methods. Bars show the mean ± SEM. Findings in arthritic wild-type AIA mice and arthritic transgenic AIA mice for single scores were compared by Mann–Whitney test. Total scores were compared between the 4 groups with adjustment for 4 parallel tests (α* = 0.0125). NS = not significant (see Figure 1 for other definitions).

Clinical findings were corroborated by corresponding histological indices of inflammation, cartilage damage and bone erosion. Inflammatory activity on day 14 after intra-articular injection was similar in tg AIA mice when compared to WT AIA mice (Figure 2A, B, and C). Similarly, cartilage degradation and proteoglycan loss in the knee joints were not different in tg AIA mice when compared to WT AIA mice (Figures 2D, E, and F). There was no significant difference between tg and WT AIA mice, neither in the single histological scores for synovitis, joint space exudate, soft tissue inflammation, cartilage degradation/proteoglycan loss, and bone erosion, nor in the total histology score (Figures 2G and H).

#### Micro-CT

**Figure 3:**
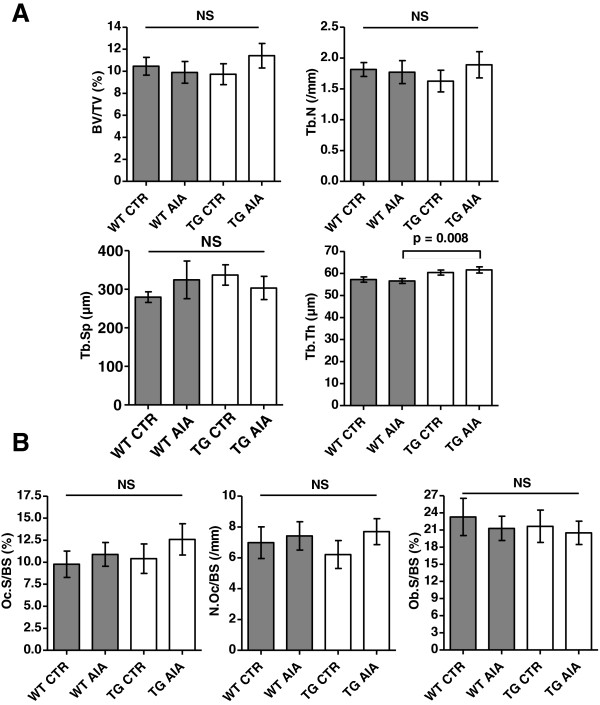
**Micro-CT and histomorphometric analysis of the contralateral proximal tibia 14 days post injection of mBSA.** Bone turnover was measured at a location distant to the site of active inflammation to assess the systemic effects of joint inflammation. **(A)** Micro-CT. The bone volume fraction (bone volume/tissue volume (BV/TV]), trabecular number (Tb.N), trabecular separation (Tb.Sp), and trabecular thickness (Tb.Th) are shown for bones harvested. Bars show the mean ± SEM. Findings between the 4 groups were compared using the Mann–Whitney-Test with adjustment for 4 parallel tests (α* = 0.0125). NS = not significant (see Figure 1 for other definitions). **(B)** Histomorphometric quantification. Bone resorption, shown as osteoclast surface/bone surface (Oc.S/BS) and osteoclast number/bone surface (N.Oc/BS), respectively. Bone formation, shown as osteoblast surface/bone surface (Ob.S/BS). Bars show the mean ± SEM. Findings between the 4 groups were compared by using the Mann–Whitney test with adjustment for 4 parallel tests (α* = 0.0125). NS = not significant (see Figure 1 for other definitions).

To quantify the *systemic* effects of inflammation on bone metabolism, the contralateral (left) proximal tibia (i.e., a skeletal region remote from the actual site of inflammation) was analysed by micro-CT. No statistically significant difference was detectable between arthritic and control mice, as well as between tg and WT mice in terms of the bone volume fraction (bone volume/tissue volume [BV/TV]), trabecular number and trabecular separation. This indicates that locally restricted AIA does not affect bone morphology systemically, at least not after 14 days. There was a statistical difference in trabecular thickness with higher values in tg than in WT arthritic mice (Figure 3A).

#### Bone histomorphometry

Histomorphometry was performed at the contralateral (left) proximal tibiae of mice. Mean osteoclast surface per bone surface and mean osteoclast number per bone surface as well as mean osteoblast surface per bone surface were not significantly different in arthritic versus non-arthritic mice, nor was there any difference seen between tg and WT mice (Figure 3B). This indicates that short-term, monarticular inflammation has little or no effect on systemic bone turnover.

#### Serum levels of cytokines

The serum levels of 32 cytokines, chemokines and growth factors were determined in AIA and control mice. There was a statistically significant difference between AIA WT and tg mice for IL-1α, IL-12 (p70) and IL-13 on day 14. However, a statistically significant difference between arthritic and control mice was not detectable (Table 1). There were no significant differences in TNF-α, IL-1β, IL-9, IL-10, IL-12 (p40), IL-15, IL-18, MCP-1, MIP-1α, MIP-1β, RANTES, KC, MIG, Eotaxin, LIF, basic-FGF, PDGF-BB, VEGF, M-CSF or G-CSF levels between WT and tg mice, or between AIA and control mice. Serum concentrations of IFN-γ, IL-2, IL-3, IL-4, IL-5, IL-6, IL-17A, MIP-2, and GM-CSF were below the detection limit in the majority of mice.

**Table 1:** Serum cytokine levels in control mice and arthritic AIA mice

**Cytokine**	**Control mice, day 14**		**AIA mice, day 14**	
	**Wild-type (n = 15)**	**Transgenic (n = 14)**	**Wild-type (n = 17)**	**Transgenic (n = 17)**
TNF-α (pg/ml)	36 (36–408)	36 (36–36)	409 (36–476)	36 (36–341)
IL-1α (pg/ml)	67 (58–71)	5 (5–64)	89 (54–97)	44 (5–66)#
IL-1β (pg/ml)	558 (46–1,050)	46 (46–1,097)	574 (46–1,397)	46 (46–431)
IL-6 (pg/ml)	6 (6–6)	6 (6–6)	6 (6–6)	6 (6–6)
IL-10 (pg/ml)	50 (5–111)	5 (5–61)	72 (46–107)	50 (5–64)
IL-12 (p40) (pg/ml)	175 (114–222)	131 (114–183)	193 (147–272)	152 (130–224)
IL-12 (p70) (pg/ml)	149 (13–202)	108 (13–149)	162 (123–236)	108 (13–130)#
IL-13 (pg/ml)	45 (45–964)	45 (45–523)	852 (361–1470)	45 (45–45)#
M-CSF (pg/ml)	204 (119–593)	548 (158–612)	295 (115–481)	488 (146–608)
G-CSF (pg/ml)	121 (13–257)	59 (13–140)	165 (13–216)	105 (13–189)
**Cytokine**	**Control mice, day 28**		**AIA mice, day 28**	
	**Wild-type (n = 8)**	**Transgenic (n = 9)**	**Wild-type (n = 7)**	**Transgenic (n = 6)**
TNF-α (pg/ml)	1,361 (903–2310)	1,255 (655–1468)	1,255 (655–1468)	1,577 (848–1685)
IL-1α (pg/ml)	9 (9–9)	9 (9–9)	9 (9–9)	9 (9–9)
IL-1β (pg/ml)	14 (14–144)	14 (14–14)	14 (14–979)	517 (14–1073)
IL-6 (pg/ml)	6 (6–6)	6 (6–6)	6 (6–6)	6 (6–6)
IL-10 (pg/ml)	56 (49–68)	44 (44–54)	49 (40–54)	68 (35–130)
IL-12 (p40) (pg/ml)	98 (90–136)	70 (56–103)	110 (81–147)	83 (72–90)
IL-12 (p70) (pg/ml)	8 (8–8)	8 (8–8)	8 (8–8)	86 (8–105) §, #
IL-13 (pg/ml)	101 (8–253)	8 (8–8)	160 (113–254)	254 (66–1374) §
M-CSF (pg/ml)	95 (85–103)	106 (81–112)	86 (79–120)	88 (77–470)
G-CSF (pg/ml)	12 (12–12)	12 (12–12)	12 (12–12)	12 (12–12)

Values indicated are the median values (interquartile range). Findings between groups were compared using the Kruskal-Wallis test, followed by multiple two-group comparisons. The Bonferroni correction was used to adjust the significance level for 4 parallel comparisons (α* = 0.0125). TNF-α = tumour necrosis factor α; IL-1α = interleukin-1α; M-CSF = macrophage colony-stimulating factor; G-CSF = granulocyte colony-stimulating factor.

Wild-type versus transgenic CTR mice: none.

^#^Wild-type versus transgenic AIA mice: IL-1α p_14_ = 0.008; IL-12 (p70) p_14_ = 0.010 and p_28_ = 0.009; IL-13 p_14_ = 0.001.

CTR versus AIA wild-type mice: none.

^§^CTR versus AIA transgenic mice: IL-12 (p70) p_28_ = 0.001; IL-13 p_28_ = 0.008.

### Prolonged arthritis

#### Body weight

Since in acute AIA, arthritis tended to resolve from day 2 p.i. onwards, we further tested our hypothesis in a model of prolonged antigen exposure, where inflammation was maintained for up to 28 days through weekly injections of mBSA. Body weight at baseline was significantly higher in WT (mean ± SD: 38.6 ± 3.4 g) compared to tg mice (36.1 ± 1.4 g; *P* = 0.016). After each flare-up reaction, a minor weight loss (<10%) was observed in both arthritic and control mice [*P* = 0.314]. The degree of weight loss was similar in arthritic WT and tg mice [*P*_AIA_ = 0.553].

#### Knee joint swelling

Knee diameter prior to induction of arthritis (day 0) was not different between WT and tg mice (mean ± SD 3.88 ± 0.07 mm and 3.85 ± 0.08 mm, respectively; *P* = 0.203). As observed in acute AIA, acute arthritis with significant knee joint swelling in comparison to controls developed in both WT and tg mice in response to the initial injection of mBSA [*P* < 0.001]. Maximum knee joint swelling was observed on day 1 post injection. Further flare-up reactions were observed on days 8, 15 and 22. However, there was no statistically significant difference in knee joint swelling between arthritic WT and tg mice over the observation period of 28 days [*P*_AIA_ = 0.456] (Figure 1B).

#### Histopathological assessment of inflamed joints

**Figure 4:**
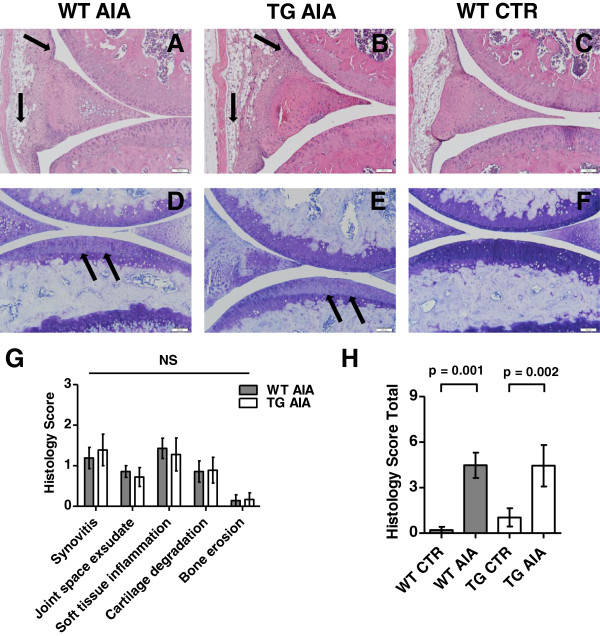
**Arthritis and cartilage damage in the knee joint on day 28 of prolonged arthritis.** Mice received intra-articular injection on day 0 and three repeated intravenous boosts with antigen or PBS on days 7, 14, 21. **(A–F)** Representative histologic sections of knee joints from wild-type (WT) and transgenic (TG) mice treated with mBSA (AIA) and from non-arthritic wild-type control mice (CTR). Both inflammatory activity and cartilage damage were similar in TG and WT mice. (**A–C)** Haematoxylin and eosin staining. **Arrows** show synovitis and soft tissue inflammation. Bars = 100 μm. **(D–F)** Toluidine blue staining. **Arrows** show proteoglycan loss of articular cartilage. Bars = 100 μm. **(G-H)** Histopathology scores (G single, H total) in mBSA treated mice (AIA) on day 28 post intra-articular injection and three flare-up reactions. The knee joints of all mice were assessed as described in Materials and Methods. Bars show the mean ± SEM. Findings in arthritic wild-type and transgenic mice for single scores were compared by Mann–Whitney test. Total scores were compared between the 4 groups with adjustment for 4 parallel tests (α* = 0.0125). NS = not significant.

Histological inflammatory activity, cartilage degradation and bone erosion, as assessed on day 28, were not seen to be different between tg and WT AIA mice (Figure 4).

#### Micro-CT

In micro-CT analysis of the contralateral tibia we found no statistically significant differences between arthritic and control mice regardless of the genotypes in terms of BV/TV (WT CTR 10.1 ± 2.0%; WT AIA 10.6 ± 1.6%; tg CTR 9.3 ± 3.1%; tg AIA 8.7 ± 2.8%), trabecular number (WT CTR 1.649 ± 0.405/mm; WT AIA 1.861 ± 0.287/mm; tg CTR 1.554 ± 0.490/mm; tg AIA 1.345 ± 0.435/mm) and trabecular separation (WT CTR 342.6 ± 123.7 μm; WT AIA 261.5 ± 28.8 μm; tg CTR 337.1 ± 85.8 μm; tg AIA 392.8 ± 125.6 μm) after repeated flare-up reactions, except for a higher trabecular thickness in tg than in WT AIA mice (tg AIA 64.5 ± 4.5 μm vs. WT AIA 57.0 ± 4.1 μm, p = 0.005; WT CTR 62.2 ± 5.8 μm; tg CTR 59.8 ± 3.1 μm).

#### Bone histomorphometry

Histomorphometric analysis of the contralateral tibia demonstrated no statistically significant differences in terms of osteoclast number per bone surface (WT CTR 4.8 ± 1.6/mm; WT AIA 7.7 ± 2.6/mm; tg CTR 7.2 ± 1.7/mm; tg AIA 6.0 ± 2.1/mm) and osteoblast surface per bone surface (WT CTR 14.4 ± 3.6%; WT AIA 14.5 ± 5.0%; tg CTR 16.8 ± 3.6%; tg AIA 17.1 ± 5.7%) after repeated flare-up reactions, except for a higher mean osteoclast surface per bone surface in tg control mice than in WT control mice (tg CTR 9.4 ± 2.6% vs. WT CTR 5.8 ± 1.7%, p = 0.009; WT AIA 9.6 ± 3.9%; tg AIA 7.2 ± 2.4%).

#### Serum levels of cytokines

There was a statistically significant difference between AIA WT and tg mice for IL-12 (p70) on day 28 (Table 1). However, a statistically significant arthritis effect was detectable for IL-12 and IL-13 levels between tg AIA and control mice only, whereas in WT mice there was no difference between arthritic and non-arthritic mice. There were no significant differences in TNF-α, IL-1β, IL-9, IL-10, IL-12 (p40), IL-15, IL-18, MIP-1α, MIP-1β, RANTES, KC, MIG, Eotaxin, basic-FGF, PDGF-BB, VEGF or M-CSF levels between WT and tg mice, or between AIA and control mice. Serum concentrations of IFN-γ, IL-2, IL-3, IL-4, IL-5, IL-6, IL-17A, MIP-2, GM-CSF and MCP-1, LIF and G-CSF were below the detection limit in the majority of mice.

## Discussion

In sharp contrast to our previous findings in K/BxN serum-induced arthritis [8], the present study demonstrates that murine antigen-induced arthritis (AIA) is unaffected by disruption of GC signalling in osteoblasts. The most important variables for the decision to reject the hypothesis were clinical signs and histology. Neither in acute nor in prolonged AIA did any clinical signs of arthritis differ significantly between transgenic and WT mice. The impression that the WT- and tg-mice in the short-time and long-time experiments react differently to arthritis induction (Figure 1) is due to the lower number of animals in the long-time experiments. This was confirmed via histological assessment of inflammatory activity, cartilage degradation or bone erosion, where also no significant differences were detectable in both experiments.

The immunological and inflammatory profiles of AIA differ profoundly from those of K/BxN arthritis (Table 2). In AIA, disruption of GC-signalling in osteoblasts is not relevant, probably because the antibody- mediated inflammatory response is of minor importance and the T cell response overcomes other effects. Antigen-specific T cells, in particular CD4+ T helper cells, generated within the adaptive immune response initiated through immunisation, play a major role in AIA as arthritis in this model cannot be induced in T cell-deficient mice [21,22,30]. The antibody-mediated inflammatory response is present in AIA as well, but apparently this is of minor importance here. This is evidenced by the observations that (i) mBSA antibodies-containing serum can only induce very weak arthritis and (ii) antibody-producing B cells are not at all required for induction of arthritis [21,22,30]. The mBSA challenge induces a significant neutrophil migration which peaks at 24 hours and subsides by seven days after challenge [31,32]. Fc Receptors are involved in this activation [33,34], but – in contrast to exclusively antibody-mediated arthritis models – complement appears to play a minor role. This conclusion has been derived from findings which demonstrate that flare-up reactions of AIA are complement-independent, and that neutrophils occur in the synovial tissue of complement-depleted mice as well [35].

**Table 2:** Immunological and inflammatory profiles of AIA and K/BxN models

**Compartment**	**Antigen-induced arthritis (AIA)**	**K/BxN serum-induced arthritis (KRN)**
T cells	T cell-dependent [21,22,30]	T cell-independent [9]
B cells	B cell-independent [22]	B cell-independent [9]
Antibodies	Antibodies unnecessary/insufficient to induce arthritis to the full extent [21,22,30]	Antibodies necessary/sufficient to induce arthritis in full extent [9]
Complement	Complement-independent [31]	Complement-dependent [12]
Fc receptors	FcγR important [32,33]	FcγR-dependent [12]
Neutrophils, macrophages	Neutrophils important [34,35], but complement-independent [31]	Neutrophil-dependent, macrophage-dependent [13,14]
Cytokines	IL-1, TNF-α, IL-6, IL-17, RANKL important [22,36]	IL-1-, TNF-α-dependent [16] IL-6-, osteopontin-independent [16,37]

In contrast, in antibody-mediated arthritis, such as K/BxN serum-induced arthritis, T cells are not required. Arthritis is provoked by the antibodies even if the recipients are devoid of lymphocytes [9,10]. Immune complexes of arthritogenic auto-antibodies act through Fc receptors and the complement network [11,12], activating neutrophils and macrophages which have been shown to be essential for induction of K/BxN serum-induced arthritis (whereas mast cells have recently been found to be apparently not mandatory) [13-15,38]. Therefore, our results suggest that the GC-dependent pathway by which osteoblasts modulate the inflammatory response is T cell-independent. Osteoblasts appear to impact the immune complex-mediated inflammatory response via a GC-dependent pathway.

The alternative complement pathway is essential in K/BxN serum-induced arthritis [12]. Complement factors are mainly generated in the liver. However, osteoblasts have been shown to express complement factors [39], and in endothelial cells, complement factor expression has been found to be GC-dependent [40]. Hence, osteoblasts may produce alternative complement factors in a GC-dependent way (e.g. C3, factor B, factor H), and reduction of these osteoblastic alternative complement factors could be responsible for attenuation of arthritis in Col2.3-11β-HSD2-transgenic mice. Other pro-inflammatory factors in antibody-mediated arthritis – which may be generated by osteoblasts in a GC-dependent way – are urokinase-type plasminogen activator (u-PA) [41-43], matrix metalloproteinases (MMP) [18,43] and macrophage migration inhibitory factor (MIF) [44-46]. Osteopontin and IL-6 are rather unlikely to be responsible because they play no essential role in K/BxN arthritis [16,37].

We found no differences in multiplex analysis of serum cytokine levels between arthritic and control mice for WT mice, and only IL-1α, IL-12 p40 and IL-13 for tg mice. This is in accordance with a recently published follow up (from 7 hours to 14 days) multiplex analysis of 24 cytokines in synovial fluid and sera of rats developing antigen-induced arthritis [47]. Cytokine concentrations in sera also showed only little variation here, whereas between cytokine concentrations in arthritic synovial fluid and histological or clinical parameters some correlations were established [47]. We agree with the authors, that “such results are consistent with the local and monarticular nature of AIA, rendering the amount of cytokines produced within a single diseased joint prone to be reduced by degradation in the lymphatic system or by dilution into the bloodstream or both” [47]. The relevance of the differences for IL-1α, IL-12 p40 and IL-13 between AIA WT and tg mice remains unclear. IL-12 p40 and IL-13 levels were lower in tg mice in comparison to WT mice after 14 days, but paradoxically higher after 28 days. In our study of K/BxN serum-induced arthritis, IL-12 p40 levels had not been different, and IL-6 and M-CSF levels tended to be altered in tg mice in comparison to WT mice (IL-1α and IL-13 levels were not determined) [8]. There is indeed evidence for a (endogenous) glucocorticoid modulation of IL-1α, IL-12 p40 and IL-13 [48-51]; however, such a modulation in osteoblasts apparently is of no relevance for AIA.

We did not measure a decrease of BV/TV or an increase in osteoclast covered bone surface due to AIA-arthritis in WT-mice compared to the control group of WT-mice, probably because μCT and histomorphometric measurements were done at the contralateral tibia. Even if the severity of arthritis at the arthritic knee in this model reached a normal extent, comparable to what is known in literature [52], apparently there are no systemic effects on bone due to the monarticular nature of AIA. This is in line with the cytokine measurements (see above). In our studies of K/BxN arthritis we had seen a systemic effect of arthritis on bone in μCT and histomorphometry at the tibia in WT mice, which was prevented in tg mice [8]. The extent of erosions achieved in the arthritic knee in AIA was low in short and long-term experiments, consistent with previous investigations [53,54].

## Conclusions

To summarise, murine AIA is not affected by disruption of GC signalling in osteoblasts. Our findings suggest that osteoblasts do not modulate the T cell-mediated inflammatory response via a GC-dependent pathway.

## Abbreviations

AIA: Antigen-induced arthritis; basic-FGF: Basic fibroblast growth factor; BV/TV: Bone volume/tissue volume; CFA: Freund’s complete adjuvant; Col2.3: 2.3-kb collagen type Iα1 promotor; CTR: Control(s); EDTA: Ethylenediaminetetraacetic acid; GC: Glucocorticoid(s); G-CSF: Granulocyte colony-stimulating factor; GM-CSF: Granulocyte macrophage colony-stimulating factor; H&E: Haematoxylin and eosin; 11β-HSD2: 11β-hydroxysteroid dehydrogenase type 2; IL: Interleukin; IFN-γ: Interferon-γ; KC: Keratinocyte-derived cytokine; LIF: Leukaemia inhibitory factor; M-CSF: Macrophage colony-stimulating factor; mBSA: Methylated bovine serum albumine; MCP-1: Monocyte chemotactic protein-1; micro-CT: Microfocal-computed tomography; MIF: Macrophage migration inhibitory factor; MIG: Monokine-induced by interferon-gamma; MIP-1α: Macrophage inflammatory protein 1α; MMP: Matrix metalloproteinases; N.Oc/BS: Osteoclast number/bone surface; Ob.S/BS: Osteoblast surface/bone surface; Oc.S/BS: Osteoclast surface/bone surface; PBS: Phosphate-buffered saline; PDGF-BB: Platelet-derived growth factor homodimer; RA: Rheumatoid arthritis; RANKL: Receptor activator of nuclear factor kappa-B ligand; RANTES: Regulated upon activation normal T cell expressed and secreted; SD: Standard deviation; SEM: Standard error of the mean; Tb.N: Trabecular number; Tb.Sp: Trabecular separation; Tb.Th: Trabecular thickness; tg: Transgenic; TRAP: Tartrate-resistant acid phosphatase; TNF-α: Tumour necrosis factor α; u-PA: Urokinase-type plasminogen activator; VEGF: Vascular endothelial growth factor; WT: Wild-type.

## Competing interests

The authors declare that they have no competing interests.

## Authors’ contributions

CS planned and performed experiments, analysed and interpreted data, performed the histological scoring and drafted the manuscript; EW performed experiments, analysed and interpreted data, performed the histological scoring and carried out the micro-CT and histomorphometry analysis; JWT did experiments and performed the histological scoring; AL did pre-experiments; TG carried out the cytokine analysis and planned the study; DH performed the statistical analysis; HZ, MJS and FB supervised the project, planned the study and reviewed the manuscript. All authors read and approved the final manuscript.

## Pre-publication history

The pre-publication history for this paper can be accessed here:

http://www.biomedcentral.com/1471-2474/15/31/prepub

## References

[B1] Buttgereit F Zhou H Seibel MJ Arthritis and endogenous glucocorticoids: the emerging role of the 11beta-HSD enzymes Ann Rheum Dis 2008 67 9 1201 1203 18697779 10.1136/ard.2008.092502

[B2] Hardy R Rabbitt EH Filer A Emery P Hewison M Stewart PM Gittoes NJ Buckley CD Raza K Cooper MS Local and systemic glucocorticoid metabolism in inflammatory arthritis Ann Rheum Dis 2008 67 9 1204 1210 18420938 10.1136/ard.2008.090662 PMC2564803

[B3] Straub RH Dhabhar FS Bijlsma JW Cutolo M How psychological stress via hormones and nerve fibers may exacerbate rheumatoid arthritis Arthritis Rheum 2005 52 1 16 26 10.1002/art.20747 15641084

[B4] Saldanha C Tougas G Grace E Evidence for anti-inflammatory effect of normal circulating plasma cortisol Clin Exp Rheumatol 1986 4 4 365 366 3791721

[B5] Coutinho AE Gray M Brownstein DG Salter DM Sawatzky DA Clay S Gilmour JS Seckl JR Savill JS Chapman KE 11beta-Hydroxysteroid dehydrogenase type 1, but not type 2, deficiency worsens acute inflammation and experimental arthritis in mice Endocrinology 2012 153 1 234 240 10.1210/en.2011-1398 22067318 PMC3279737

[B6] Ergang P Leden P Vagnerova K Klusonova P Miksik I Jurcovicova J Kment M Pacha J Local metabolism of glucocorticoids and its role in rat adjuvant arthritis Mol Cell Endocrinol 2010 323 2 155 160 10.1016/j.mce.2010.03.003 20226838

[B7] Wilckens T Volkmann A Cortisol metabolism by 11 beta-hydroxysteroid dehydrogenase as a novel target in the treatment of inflammation- or immune-mediated bone loss: comment on the article by Makrygiannakis et al Arthritis Rheum 2007 56 1 387 388 10.1002/art.22318 17195247

[B8] Buttgereit F Zhou H Kalak R Gaber T Spies CM Huscher D Straub RH Modzelewski J Dunstan CR Seibel MJ Transgenic disruption of glucocorticoid signaling in mature osteoblasts and osteocytes attenuates K/BxN mouse serum-induced arthritis in vivo Arthritis Rheum 2009 60 7 1998 2007 10.1002/art.24619 19565501

[B9] Korganow AS Ji H Mangialaio S Duchatelle V Pelanda R Martin T Degott C Kikutani H Rajewsky K Pasquali JL From systemic T cell self-reactivity to organ-specific autoimmune disease via immunoglobulins Immunity 1999 10 4 451 461 10.1016/S1074-7613(00)80045-X 10229188

[B10] Nandakumar KS Backlund J Vestberg M Holmdahl R Collagen type II (CII)-specific antibodies induce arthritis in the absence of T or B cells but the arthritis progression is enhanced by CII-reactive T cells Arthritis Res Ther 2004 6 6 R544 550 10.1186/ar1217 15535832 PMC1064861

[B11] Mancardi DA Jonsson F Iannascoli B Khun H Van Rooijen N Huerre M Daeron M Bruhns P Cutting edge: the murine high-affinity IgG receptor FcgammaRIV is sufficient for autoantibody-induced arthritis J Immunol 2011 186 4 1899 1903 10.4049/jimmunol.1003642 21248252

[B12] Ji H Ohmura K Mahmood U Lee DM Hofhuis FM Boackle SA Takahashi K Holers VM Walport M Gerard C Arthritis critically dependent on innate immune system players Immunity 2002 16 2 157 168 10.1016/S1074-7613(02)00275-3 11869678

[B13] Solomon S Rajasekaran N Jeisy-Walder E Snapper SB Illges H A crucial role for macrophages in the pathology of K/B x N serum-induced arthritis Eur J Immunol 2005 35 10 3064 3073 10.1002/eji.200526167 16180250

[B14] Wipke BT Allen PM Essential role of neutrophils in the initiation and progression of a murine model of rheumatoid arthritis J Immunol 2001 167 3 1601 1608 11466382 10.4049/jimmunol.167.3.1601

[B15] Monach PA Nigrovic PA Chen M Hock H Lee DM Benoist C Mathis D Neutrophils in a mouse model of autoantibody-mediated arthritis: critical producers of Fc receptor gamma, the receptor for C5a, and lymphocyte function-associated antigen 1 Arthritis Rheum 2010 62 3 753 764 10.1002/art.27238 20191628 PMC3057458

[B16] Ji H Pettit A Ohmura K Ortiz-Lopez A Duchatelle V Degott C Gravallese E Mathis D Benoist C Critical roles for interleukin 1 and tumor necrosis factor alpha in antibody-induced arthritis J Exp Med 2002 196 1 77 85 10.1084/jem.20020439 12093872 PMC2194010

[B17] Terauchi M Li JY Bedi B Baek KH Tawfeek H Galley S Gilbert L Nanes MS Zayzafoon M Guldberg R T lymphocytes amplify the anabolic activity of parathyroid hormone through Wnt10b signaling Cell Metab 2009 10 3 229 240 10.1016/j.cmet.2009.07.010 19723499 PMC2751855

[B18] Zhou H Mak W Kalak R Street J Fong-Yee C Zheng Y Dunstan CR Seibel MJ Glucocorticoid-dependent Wnt signaling by mature osteoblasts is a key regulator of cranial skeletal development in mice Development 2009 136 3 427 436 10.1242/dev.027706 19141672

[B19] Stanley KT VanDort C Motyl C Endres J Fox DA Immunocompetent properties of human osteoblasts: interactions with T lymphocytes J Bone Miner Res 2006 21 1 29 36 16355271 10.1359/JBMR.051004

[B20] Swolin-Eide D Ohlsson C Effects of cortisol on the expression of interleukin-6 and interleukin-1 beta in human osteoblast-like cells J Endocrinol 1998 156 1 107 114 10.1677/joe.0.1560107 9496240

[B21] Brackertz D Mitchell GF Mackay IR Antigen-induced arthritis in mice. I. Induction of arthritis in various strains of mice Arthritis Rheum 1977 20 3 841 850 10.1002/art.1780200314 857805

[B22] Wong PK Quinn JM Sims NA van Nieuwenhuijze A Campbell IK Wicks IP Interleukin-6 modulates production of T lymphocyte-derived cytokines in antigen-induced arthritis and drives inflammation-induced osteoclastogenesis Arthritis Rheum 2006 54 1 158 168 10.1002/art.21537 16385511

[B23] Sher LB Woitge HW Adams DJ Gronowicz GA Krozowski Z Harrison JR Kream BE Transgenic expression of 11beta-hydroxysteroid dehydrogenase type 2 in osteoblasts reveals an anabolic role for endogenous glucocorticoids in bone Endocrinology 2004 145 2 922 929 10.1210/en.2003-0655 14617568

[B24] Zhou H Mak W Zheng Y Dunstan CR Seibel MJ Osteoblasts directly control lineage commitment of mesenchymal progenitor cells through Wnt signaling J Biol Chem 2008 283 4 1936 1945 18045882 10.1074/jbc.M702687200

[B25] Kalak R Zhou H Street J Day RE Modzelewski JR Spies CM Liu PY Li G Dunstan CR Seibel MJ Endogenous glucocorticoid signalling in osteoblasts is necessary to maintain normal bone structure in mice Bone 2009 45 1 61 67 10.1016/j.bone.2009.03.673 19358901

[B26] van den Berg WB Joosten LA van Lent PL Murine antigen-induced arthritis Methods Mol Med 2007 136 243 253 10.1007/978-1-59745-402-5_18 17983153

[B27] Yang YH Morand EF Getting SJ Paul-Clark M Liu DL Yona S Hannon R Buckingham JC Perretti M Flower RJ Modulation of inflammation and response to dexamethasone by annexin 1 in antigen-induced arthritis Arthritis Rheum 2004 50 3 976 984 10.1002/art.20201 15022342

[B28] Lens JW van den Berg WB van de Putte LB Flare-up of antigen-induced arthritis in mice after challenge with intravenous antigen: studies on the characteristics of and mechanisms involved in the reaction Clin Exp Immunol 1984 55 2 287 294 6199138 PMC1535823

[B29] Morgan E Varro R Sepulveda H Ember JA Apgar J Wilson J Lowe L Chen R Shivraj L Agadir A Cytometric bead array: a multiplexed assay platform with applications in various areas of biology Clin Immunol 2004 110 3 252 266 10.1016/j.clim.2003.11.017 15047203

[B30] Brackertz D Mitchell GF Vadas MA Mackay IR Studies on antigen-induced arthritis in mice. III. Cell and serum transfer experiments J Immunol 1977 118 5 1645 1648 300753

[B31] Lens JW van den Berg WB van de Putte LB Berden JH Lems SP Flare-up of antigen-induced arthritis in mice after challenge with intravenous antigen: effects of pre-treatment with cobra venom factor and anti-lymphocyte serum Clin Exp Immunol 1984 57 3 520 528 6235993 PMC1536280

[B32] van Lent PL Nabbe K Blom AB Holthuysen AE Sloetjes A van de Putte LB Verbeek S van den Berg WB Role of activatory Fc gamma RI and Fc gamma RIII and inhibitory Fc gamma RII in inflammation and cartilage destruction during experimental antigen-induced arthritis Am J Pathol 2001 159 6 2309 2320 10.1016/S0002-9440(10)63081-7 11733380 PMC1850614

[B33] van Lent PL van Vuuren AJ Blom AB Holthuysen AE van de Putte LB van de Winkel JG van den Berg WB Role of Fc receptor gamma chain in inflammation and cartilage damage during experimental antigen-induced arthritis Arthritis Rheum 2000 43 4 740 752 10.1002/1529-0131(200004)43:4<740::AID-ANR4>3.0.CO;2-0 10765918

[B34] Grespan R Fukada SY Lemos HP Vieira SM Napimoga MH Teixeira MM Fraser AR Liew FY McInnes IB Cunha FQ CXCR2-specific chemokines mediate leukotriene B4-dependent recruitment of neutrophils to inflamed joints in mice with antigen-induced arthritis Arthritis Rheum 2008 58 7 2030 2040 10.1002/art.23597 18576322

[B35] Coelho FM Pinho V Amaral FA Sachs D Costa VV Rodrigues DH Vieira AT Silva TA Souza DG Bertini R The chemokine receptors CXCR1/CXCR2 modulate antigen-induced arthritis by regulating adhesion of neutrophils to the synovial microvasculature Arthritis Rheum 2008 58 8 2329 2337 10.1002/art.23622 18668539

[B36] Koenders MI Lubberts E Oppers-Walgreen B van den Bersselaar L Helsen MM Di Padova FE Boots AM Gram H Joosten LA van den Berg WB Blocking of interleukin-17 during reactivation of experimental arthritis prevents joint inflammation and bone erosion by decreasing RANKL and interleukin-1 Am J Pathol 2005 167 1 141 149 10.1016/S0002-9440(10)62961-6 15972960 PMC1603454

[B37] Jacobs JP Pettit AR Shinohara ML Jansson M Cantor H Gravallese EM Mathis D Benoist C Lack of requirement of osteopontin for inflammation, bone erosion, and cartilage damage in the K/BxN model of autoantibody-mediated arthritis Arthritis Rheum 2004 50 8 2685 2694 10.1002/art.20381 15334485

[B38] Feyerabend TB Weiser A Tietz A Stassen M Harris N Kopf M Radermacher P Moller P Benoist C Mathis D Cre-mediated cell ablation contests mast cell contribution in models of antibody- and T cell-mediated autoimmunity Immunity 2011 35 5 832 844 10.1016/j.immuni.2011.09.015 22101159

[B39] Ignatius A Schoengraf P Kreja L Liedert A Recknagel S Kandert S Brenner RE Schneider M Lambris JD Huber-Lang M Complement C3a and C5a modulate osteoclast formation and inflammatory response of osteoblasts in synergism with IL-1beta J Cell Biochem 2011 112 9 2594 2605 10.1002/jcb.23186 21598302 PMC3158833

[B40] Coulpier M Andreev S Lemercier C Dauchel H Lees O Fontaine M Ripoche J Activation of the endothelium by IL-1 alpha and glucocorticoids results in major increase of complement C3 and factor B production and generation of C3a Clin Exp Immunol 1995 101 1 142 149 7621583 10.1111/j.1365-2249.1995.tb02290.x PMC1553312

[B41] Cook AD De Nardo CM Braine EL Turner AL Vlahos R Way KJ Beckman SK Lenzo JC Hamilton JA Urokinase-type plasminogen activator and arthritis progression: role in systemic disease with immune complex involvement Arthritis Res Ther 2010 12 2 R37 10.1186/ar2946 20196869 PMC2888184

[B42] De Nardo CM Lenzo JC Pobjoy J Hamilton JA Cook AD Urokinase-type plasminogen activator and arthritis progression: contrasting roles in systemic and monoarticular arthritis models Arthritis Res Ther 2010 12 5 R199 10.1186/ar3171 20973954 PMC2991036

[B43] Hecht M Heider U Kaiser M von Metzler I Sterz J Sezer O Osteoblasts promote migration and invasion of myeloma cells through upregulation of matrix metalloproteinases, urokinase plasminogen activator, hepatocyte growth factor and activation of p38 MAPK Br J Haematol 2007 138 4 446 458 10.1111/j.1365-2141.2007.06665.x 17593251

[B44] Santos LL Fan H Hall P Ngo D Mackay CR Fingerle-Rowson G Bucala R Hickey MJ Morand EF Macrophage migration inhibitory factor regulates neutrophil chemotactic responses in inflammatory arthritis in mice Arthritis Rheum 2011 63 4 960 970 10.1002/art.30203 21452319 PMC3069137

[B45] Onodera S Suzuki K Matsuno T Kaneda K Kuriyama T Nishihira J Identification of macrophage migration inhibitory factor in murine neonatal calvariae and osteoblasts Immunology 1996 89 3 430 435 10.1046/j.1365-2567.1996.d01-751.x 8958058 PMC1456561

[B46] Calandra T Bernhagen J Metz CN Spiegel LA Bacher M Donnelly T Cerami A Bucala R MIF as a glucocorticoid-induced modulator of cytokine production Nature 1995 377 6544 68 71 10.1038/377068a0 7659164

[B47] Paquet J Goebel JC Delaunay C Pinzano A Grossin L Cournil-Henrionnet C Gillet P Netter P Jouzeau JY Moulin D Cytokines profiling by multiplex analysis in experimental arthritis: which pathophysiological relevance for articular versus systemic mediators? Arthritis Res Ther 2012 14 2 R60 10.1186/ar3774 22414623 PMC3446427

[B48] Elenkov IJ Papanicolaou DA Wilder RL Chrousos GP Modulatory effects of glucocorticoids and catecholamines on human interleukin-12 and interleukin-10 production: clinical implications Proc Assoc Am Physicians 1996 108 5 374 381 8902882

[B49] Josephson MB Jiao J Xu S Hu A Paranjape C Grunstein JS Grumbach Y Nino G Kreiger PA McDonough J IL-13-induced changes in endogenous glucocorticoid metabolism in the lung regulate the proasthmatic response Am J Physiol Lung Cell Mol Physiol 2012 303 5 L382 390 10.1152/ajplung.00125.2012 22773690 PMC3468426

[B50] Miyazaki Y Yokozeki H Awad S Igawa K Minatohara K Satoh T Katayama I Nishioka K Glucocorticoids augment the chemically induced production and gene expression of interleukin-1alpha through NF-kappaB and AP-1 activation in murine epidermal cells J Invest Dermatol 2000 115 4 746 752 10.1046/j.1523-1747.2000.00101.x 10998154

[B51] Horiuchi Y Bae SJ Katayama I FK506 (tacrolimus) inhibition of intracellular production and enhancement of interleukin 1alpha through glucocorticoid application to chemically treated human keratinocytes Skin Pharmacol Physiol 2005 18 5 241 246 10.1159/000086670 16015023

[B52] Baschant U Frappart L Rauchhaus U Bruns L Reichardt HM Kamradt T Brauer R Tuckermann JP Glucocorticoid therapy of antigen-induced arthritis depends on the dimerized glucocorticoid receptor in T cells Proc Natl Acad Sci U S A 2011 108 48 19317 19322 10.1073/pnas.1105857108 22084093 PMC3228447

[B53] Ebbinghaus M Gajda M Boettger MK Schaible HG Brauer R The anti-inflammatory effects of sympathectomy in murine antigen-induced arthritis are associated with a reduction of Th1 and Th17 responses Ann Rheum Dis 2012 71 2 253 261 10.1136/ard.2011.150318 21953345

[B54] Imhof AK Gluck L Gajda M Brauer R Schaible HG Schulz S Potent anti-inflammatory and antinociceptive activity of the endothelin receptor antagonist bosentan in monoarthritic mice Arthritis Res Ther 2011 13 3 R97 10.1186/ar3372 21689431 PMC3218912

